# The dynamic interaction between symptoms and pharmacological treatment in patients with major depressive disorder: the role of network intervention analysis

**DOI:** 10.1186/s12888-023-05300-y

**Published:** 2023-11-28

**Authors:** Claudia Savia Guerrera, Giuseppe Alessio Platania, Francesco Maria Boccaccio, Pierfrancesco Sarti, Simone Varrasi, Chiara Colliva, Margherita Grasso, Simona De Vivo, Davide Cavallaro, Fabio Tascedda, Concetta Pirrone, Filippo Drago, Santo Di Nuovo, Johanna M. C. Blom, Filippo Caraci, Sabrina Castellano

**Affiliations:** 1 Department of Educational Sciences, University of Catania, Catania, Italy; 2 Department of Biomedical and Biotechnological Sciences, University of Catania, Catania, Italy; 3 Department of Biomedical, Metabolic and Neural Sciences, University of Modena and Reggio Emilia, Modena, Italy; 4 Azienda Unità Sanitaria Locale Di Modena, Distretto Di Carpi, Carpi, Italy; 5 Unit of Neuropharmacology and Translation Neurosciences, Oasi Research Institute - IRCCS, Troina, Italy; 6 Villa dei Gerani Clinic ASP3 Catania, Catania, Italy; 7 Department of Life Sciences, University of Modena and Reggio Emilia, Modena, Italy; 8 Center for Neuroscience and Neurotechnology, University of Modena and Reggio Emilia, Modena, Italy; 9 Department of Drug and Health Sciences, University of Catania, Catania, Italy

**Keywords:** Depression, Major depressive disorder, Network analysis, Antidepressants, Pharmacological treatment

## Abstract

**Introduction:**

The Major Depressive Disorder (MDD) is a mental health disorder that affects millions of people worldwide. It is characterized by persistent feelings of sadness, hopelessness, and a loss of interest in activities that were once enjoyable. MDD is a major public health concern and is the leading cause of disability, morbidity, institutionalization, and excess mortality, conferring high suicide risk. Pharmacological treatment with Selective Serotonin Reuptake Inhibitors (SSRIs) and Serotonin Noradrenaline Reuptake Inhibitors (SNRIs) is often the first choice for their efficacy and tolerability profile. However, a significant percentage of depressive individuals do not achieve remission even after an adequate trial of pharmacotherapy, a condition known as treatment-resistant depression (TRD).

**Methods:**

To better understand the complexity of clinical phenotypes in MDD we propose Network Intervention Analysis (NIA) that can help health psychology in the detection of risky behaviors, in the primary and/or secondary prevention, as well as to monitor the treatment and verify its effectiveness. The paper aims to identify the interaction and changes in network nodes and connections of 14 continuous variables with nodes identified as "Treatment" in a cohort of MDD patients recruited for their recent history of partial response to antidepressant drugs. The study analyzed the network of MDD patients at baseline and after 12 weeks of drug treatment.

**Results:**

At baseline, the network showed separate dimensions for cognitive and psychosocial-affective symptoms, with cognitive symptoms strongly affecting psychosocial functioning. The MoCA tool was identified as a potential psychometric tool for evaluating cognitive deficits and monitoring treatment response. After drug treatment, the network showed less interconnection between nodes, indicating greater stability, with antidepressants taking a central role in driving the network. Affective symptoms improved at follow-up, with the highest predictability for HDRS and BDI-II nodes being connected to the Antidepressants node.

**Conclusion:**

NIA allows us to understand not only what symptoms enhance after pharmacological treatment, but especially the role it plays within the network and with which nodes it has stronger connections.

**Supplementary Information:**

The online version contains supplementary material available at 10.1186/s12888-023-05300-y.

## Background

Major Depressive Disorder (MDD) is a mental health disorder characterized by persistent feelings of sadness, hopelessness, and a loss of interest in activities that were once enjoyable, [1] often in comorbidity with several disorders such as cardiovascular disease, dementia, and cancer [2]. In addition, a lifetime history of depression has been considered as a risk factor for later Alzheimer’s disease (AD) development and the presence of depressive symptoms can increase the conversion from mild cognitive impairment (MCI) to AD [3].

Although the prevention programs aiming at increasing awareness about potential risk factors for depression, including physical inactivity [4] and unbalanced diet [5, 6] have been proven effective, according to the Institute for Health Metrics and Evaluation (IHME), MDD affects about 3.28% of adults globally, with a peak of 4% among women, and about 4% of adults older than 60 years [7]. It has been estimated that worldwide approximately 280 million people develop depression [8].

This disorder constitute a major public health concern and is the leading cause in the global burden of disease in terms of disability, morbidity, institutionalization, especially in late-onset depression, and excess mortality, conferring high suicide risk [1, 9–12]. Depression is now widely recognised as a complex and multifactorial illness characterized by affective, cognitive and psychosocial symptoms [13, 14]. The heterogeneous nature of MDD, therefore, poses challenges to understanding the relationship linking these three different dimensions [15–17]. This highlights the need of a multimodal approach for management and treatment taking into account the complex interplay between affective, cognitive and psychosocial domains.

When considering pharmacological treatment, Selective Serotonin Reuptake Inhibitors (SSRIs) and Serotonin Noradrenaline Reuptake Inhibitors (SNRI) are the most commonly used antidepressants in MDD, often emerging as the first-choice for their efficacy and tolerability profile and ease of use [18].

The majority of antidepressant drugs have been developed according to the monoaminergic hypothesis of depression, representing a useful therapeutic tool on affective symptoms of depression, but it is unclear whether they can improve cognitive symptoms [19]. According to clinical practice guidelines, antipsychotic agents are recommended in combination with antidepressant drugs for treating depression with psychotic features or major depression with a partial response to SSRIs or SNRIs [20]. In this context, also non-pharmacological approaches such as psychotherapy [21] and physical activity were considered as add-on treatment strategies to improve cognitive deficits and affective symptoms of depression [22].

Despite the availability of multiple FDA-approved medications, including SSRIs and SNRIs, a significant percentage of depressive individuals do not achieve remission even after an adequate trial of pharmacotherapy. This condition is known as treatment-resistant depression (TRD), and its prevalence is estimated to be around 30% among MDD patients [22], probably because emerging additional factors involved in MDD pathophysiology such as the role of chronic stress and neuroinflammation, should be considered [3]. Second-generation antipsychotics (e.g. quetiapine, aripiprazole, risperidone, brexpiprazole) have been proposed in combination with SSRI/SNRIs to improve the treatment of MDD with a partial response to antidepressants (PRD) or TRD [23].

To better explain the complexity of clinical phenotypes in MDD, and the relationship between symptoms and pharmacological treatment in these patients, we propose Network Intervention Analysis, an extension of the network analysis model, which conceptualizes mental disorders as the product of interplay between symptoms. Several authors have extended the Network Analysis approach with the purpose of analyzing the specific and sequential effects of treatments on symptomatology, proposing this innovative method in the context of different psychiatric disorders [24–27]. This method, in fact, allows us both to assess the relationship between emerging symptomatology, and to consider the variable of treatment, in order to identify on which symptoms and/or variables it acts with greater effects.

More in detail, NIA analyzes the sequence of changes that the treatment induces on the symptoms and/or variables, taking into account not only the interactions among all those that are part of the network, but also specifying which among them are affected directly or indirectly by that specific treatment [28]. This method differs, hence, from traditional analyses that usually provide us only scores on severity of a disorder or dichotomous aspects, such as response or non-response to treatment [25].

The strength of NIA, therefore, is that this approach can clearly explain how a treatment is effective to improve the different symptoms and/or domains and how this effect can spread throughout the network. For all these reasons, NIA could help health psychology in the detection of risky behaviors, in the primary and/or secondary prevention, as well as to monitor the treatment and verify its effectiveness [29].

In light of the current state-of-art, this paper aims to identify the interaction and changes in network nodes and connections of 14 continuous variables with nodes identified as “Treatment” in a cohort of MDD patients recruited for their recent history of partial response to antidepressant drugs.

## Material and methods

### Setting and recruitment

Participants were enlisted from the Psychiatric Clinic "Villa dei Gerani" in Catania, Italy. Each patient was given comprehensive oral and written details regarding the intended utilization of their data and provided written consent. The study adhered to the principles outlined in the Declaration of Helsinki.

The study design was a prospective, observational (non-interventional), cohort study conducted in a clinical center in Sicily (Italy). The study complied with the definition of “observational” study (i.e., “non-interventional”) provided in Article 2(c) of Directive 2001/20/EC, meaning that the investigator who carries out the study does not interfere with the physician's decision regarding which drug is clinically pertinent to be prescribed to each individual patient [30]. Therefore, prescription of pharmacological treatments resulted solely from an independent clinical evaluation, according to the physician's clinical judgment, and based on each patient's clinical profile (presence of a depressive episode) [30].

Furthermore, the choice to include a patient in the study, after obtaining their consent, was made entirely separate from the clinical decision of prescribing psychotropic drugs. Importantly, the study had no influence on the medical practices of the participating physicians and did not lead to any extra medical appointments for the patients involved.

### Participants

Ninety MDD patients were recruited, i.e. twice the amount proposed by the initial power analysis (*n* = 44; effect size f = 0,5; alpha error probability = 0.05; power = 0,8). Nine of them were then excluded because they had not completed the psychometric protocol at T0 (first neuropsychological evaluation). The final sample at T0 was composed by eighty-one MDD patients. Twenty-eight of them (power analysis: *n* = 27; effect size f = 0,5; alpha error probability = 0,05; power = 0,8) completed 12 weeks of treatment (Table 1) and came back to Psychiatric Clinic “Villa dei Gerani” to be reassessed with the same psychometric protocol (T1 – second neuropsychological evaluation).

**Table 1:** Demographic characteristics of the overall sample at T0 and T1

Demographics	Baseline (T0)		Follow up (T1)	
	**Sample**	**Percentage**	**Sample**	**Percentage**
*Sample size*	81	100	28	100
**Gender**				
*Male*	26	32,1	10	35,7
*Female*	55	67,9	18	64,3
**Age**				
*Mean age*	53,2 ± 9,7		55,4 ± 7,9	

The criteria for inclusion in the study were:

1. A diagnosis of MDD according to DSM-5 criteria.
2. Age between 18–65 years old.
3. A recent history in MDD patients (in the last 4 weeks) of partial response to a previous treatment with an antidepressant drug.
4. Not participating in another study simultaneously.
5. Having signed an informed consent.

Criteria for exclusion from the study were:

1. A history of mental retardation or any clinical condition that could affect cognitive performance.
2. Comorbidiy with psychotic disorder.
3. Electroconvulsive therapy 1 year prior to neuropsychological assessment.

### Pharmacological treatment

At the beginning of the study (T0), each of eighty-one patients received a tailored pharmacological treatment. Although only twenty-eight of them completed 12-weeks pharmacological treatment prescribed and were retested with the same psychometric protocol (T1).

All twenty-eight patients with MDD were treated with Antidepressants and adjunctive Second Generation Antipsychotics, because they were partial responders to previous pharmacological treatments. Partial response was defined as patients not meeting remission criteria but experiencing an improvement in symptoms.

The following drugs were used: escitalopram (10 mg/day), paroxetine (20 mg/day), sertraline (100 mg/day), citalopram (40 mg/day); duloxetine (60 mg/day), venlafaxine (150–225 mg/day); risperidone (2–3 mg/day), olanzapine (2,5–5 mg/day), aripiprazole (2,5–5 mg/day).

### Neuropsychological assessment

During the observational study, patients underwent a complete neuropsychological evaluation carried out at baseline and at the end of 12-weeks of pharmacological treatment. The neuropsychological assessment, consisting of the tests reported below, at both T0 and T1 was performed within the psychiatric clinic 'Villa dei Gerani' by psychologists qualified in test administration.

#### Affective domain

- Hamilton Depression Rating Scale (HDRS) – Italian version [31]: it is a 21-item hetero-administered scale in which determinant areas are explored in assessing the subject's depressive state. A score < 7 indicates no depression; between 8 and 17 indicates mild depression; between 18 and 24 moderate depression; > 24 severe depression.
- Beck Depression Inventory (BDI–II) – Italian version [32]: it is a 21-item self-administered instrument to detect the severity of depression in adults and adolescents from age 13 onward. Scores 0–13 indicate no depressive content; scores between 14–19: mild depression; scores 20–29 moderate depression; scores 30–63: severe depression. The Italian validation data confirm the existence of two sides of depression, the mental and the somatic, as in the original edition. The internal consistency calculated through Cronbach's alpha results in 0.86 for the first factor and 0.65 for the second factor.

For both instruments, the higher the score, the worse the depressive symptomatology.

#### Neurocognitive domain

##### Global cognitive functions assessment

- Montreal Cognitive Assessment (MoCA) – Italian version [33]: it is a rapid screening tool for global cognitive functions, and executive functions. It assesses several cognitive domains: attention and concentration, executive functions, memory, language, visuoconstructive skills, abstraction, computation, and orientation. The maximum possible score is 30 points; a score of 26 or higher is considered normal.

##### Specific cognitive functions assessment

- Rey 15 Words Test – Italian version [34]: it assesses immediate and delayed memory span and provides an assessment on learning. The test consists of 5 presentations, with recall after 30 min, of a list of 15 words. The cut off for immediate recall is ≥ 28,53, for delay recall is ≥ 4,69.
- Forward and Backward Digit Span [35]: it assesses verbal memory span. Mean and Standard Deviation is available by age.
- Phonetic Verbal Fluency test (FAS) – Italian version [34]: it is a measure of phonemic word fluency, which is a type of verbal fluency. It assesses phonemic fluency by requesting an individual to orally produce as many words as possible that begin with the letters F, A, and S within a prescribed time frame, usually 1 min.
- the “Vocabulary” test from the WAIS-IV – Italian version [36, 37]: the examiner reads out a series of words that the subject must listen to carefully and give a definition. Assesses range and depth of knowledge, level of conceptualization processes, and language skills.
- Frontal Assessment Battery (FAB) – Italian version [38]: it is a hetero-administered tool useful for assessing certain frontal functions: conceptualization (analogies), lexical fluency, motor series, interference sensitivity, inhibitory control, and environmental dependence. Scores from 0 (test failure) to 3 (no errors) are given. Once the scores are summed, an adjustment is made for age and schooling.

#### Psychosocial domain

- Functioning Assessment Short Test (FAST) [39]: it was used as a primary outcome of psychosocial risk at the study endpoints to identify predictors for specific domains of function, such as: autonomy, occupational functioning, cognitive functioning, financial issues, interpersonal relationships, leisure. Cronbach’s alpha for the five components was 0.96, 0.88, 0.88, 0.91, 0.92, respectively, and for the total was 0.93. For this study, we only included the score of four sub-domains (autonomy, cognitive functioning, financial issues, interpersonal relationship).

### Statistical analyses

#### Descriptive and Inferential statistics

The collected data were initially analyzed qualitatively through the estimates of mean, standard deviations, and percentages to obtain general demographic information about the sample. The corrected scores (by age and schooling) of individual tests were treated as variables in the statistical and network analysis. Traditional independent t-test and parametric unidirectional analysis of variance (ANOVA) were performed to determine the difference among groups (T0 and T1) for continuous variables. Normal distribution was established by the Shapiro–Wilk test (*p* > 0.05 for normal intake). In addition, the homogeneity of variances within each group was established by Levene’s test for equal variation (*p* > 0.05 for assumption of equal variance), and when violated, Welch’ correction for unequal variances was applied. All analyses were conducted using SPSS version 26.0 (SPSS Inc., Chicago, IL, USA) and R software (version 4.0.3 / 2020–10-10).

#### Network analysis

Network analysis was performed on 81 MDD patients at T0 and on 28 patients that completed a 12-week treatment (T1).

Networks were computed with the package “qgraph” [40] in the R software using the Fruchterman-Reingold algorithm, which transforms the network into a system of massive particles. Nodes are interpreted as particles and edges as mutual pushes. The algorithm attempts to minimize the used energy of the physical system. Fruchterman and Reingold's (1991) algorithm [41] adds "uniform vertex distribution" compared to earlier versions.

Introducing drug treatment as a dichotomous variable (presence/absence), a Mixed Graphical Model (MGM) implemented through the R-package mgm [42] was used to compute the NIA. Networks are composed of nodes (circle nodes: test scores; square node: treatment) and edges. The edges represent the conditional dependence relations between variables. Thus, they indicate the association between two nodes controlled for associations with all the other nodes of the network. Green edges indicate positive associations, red edges negative associations, and gray edges partial correlations between dichotomous and continuous variables.

The thickness of an edge represents the strength of the association (thicker the edge, greater the correlation value). All the relationships represented in our model are pairwise interactions (k = 2, interactions). In addition, the resulting network consists of the estimates of the relationships between the variables taken two by two, and these relationships are controlled for by all other variables. This means that the absence of a relationship between two variables indicates that those two variables are conditionally independent given all the other variables. The difference size of nodes between T0 and T1 is explained as follows: if the test score increases, the node will be larger, vice versa if the score decreases, the node will be smaller.

The predictability of each node in the network was also calculated (i.e., nodewise predictability). This measure represents how much variance of the variable is explained by all the other variables with which it is connected. High values of predictability indicate that most of the variance of that variable can be predicted by the variables with which it has direct links. For all these reasons, predictability is an important measure when working in psychopathology. Because this measure gives us an idea of how clinically relevant connections are, it is useful to estimate the potential success of clinical interventions which could thereby effectively guide treatment selection.

For continuous variables, the proportion of explained variance (i.e., R2) was chosen as the measure of predictability: a value of 0 means that the node is not predicted by all neighboring nodes (i.e. all the nodes with which it has connections) in the network, while a value of 1 means that the node can be perfectly predicted by its neighboring nodes.

We analyzed two main measures of centrality: strength centrality and betweenness.

Strength centrality refers to the number of connections a node has: more connections indicate greater importance of the node in the network. In a clinical context, a symptom with many connections in a psychopathological system may be considered a risk factor for the development of other symptoms, while a symptom with fewer connections may be considered more peripheral and less likely to promote worsening of other symptoms. In weighted networks, as in this study, links connecting nodes are no longer treated as binary interactions but are weighted in proportion to the strength of the correlations.

Betweenness is a parameter that measures the involvement of a node in the shortest path between two other nodes. It helps to identify which nodes are more likely to facilitate connections in the network. For example, this measure can be used to identify important domains by examining the connectivity between a patient's problems and symptoms.

The algorithm for calculating "shortest paths" is that of Dijkstra (1959) [43], implemented in R and repurposed by Opsahl, Agneessens and Skvoretz (2010) [44].

In interpreting these indices, bootstrap tests were done to analyze their stability (bootstrapped strength centrality and bootstrapped betweenness). It was done to make sure that central nodes were also so among all the subsamples of the data and whether the centrality of a node remained so in 95% of the bootstrapped subsamples.

Lastly, a cluster holding algorithm was computed in order to explore the differences in connectivity structure among the three groups from an additional perspective. Clusters of nodes represent more connected subnetworks in a larger network. The cluster identifies a group of nodes that can be affected more rapidly when a node that is part of it changes its state. The walktrap algorithm was used to provide a measure of similarities between vertices based on random walks across the network connections (igraph package) [45] which can capture the community/cluster structure in the graph [46].

The number of clusters identified equals the number of latent factors in each dataset.

## Results

### Descriptive and inferential results

Descriptive analyses are reported for demographic data in the table above (Table 1).

Regarding the results at the psychometric tools, there are few significant differences between T0 and T1. Despite an improving trend in almost all psychological tests, only HDRS shows a significant enhancement after the 12-weeks treatment (T1) (from 23,37 to 17,92, *p* = 0.001). Regarding homogeneity of variance, Levene's Test showed significance only for HDRS (*p* = 0.014), remaining significant also after Welch’s correction (*p* = 0.007).

### Network analysis results

**Fig. 1:**
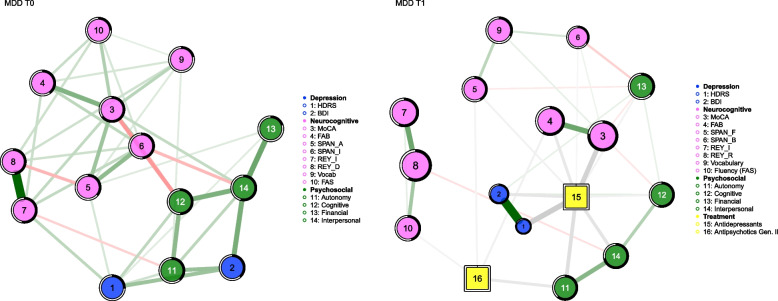
Resulting Networks in MDD sample, at baseline (T0), and after 12 weeks of treatment (T1). Left side—round nodes: continuous variables; lines between nodes: partial correlations between variables (thicker the edge, greater the correlation value); green edges: positive correlations; red edges: negative correlations; Around each node the predictability value was represented by a ring, the blacker the ring, the more predictable the variable by all connected nodes. Right side—in addition to above, square node: categorical variables; gray edge: partial correlations between dichotomous and continuous variables

At the baseline, the network of MDD (Fig. 1—left side) patients show neurocognitive and psychosocial as separated ‘dimensions’ [30]. The latter also includes the two nodes assessing depression, HDRS (1) and BDI-II (2).

Moreover, the network is well interconnected (number of edges = 39; density index = 0,42). This suggests that there is suboptimal stability because modification of a single node results in changes that easily spread to the rest of the network [47].

**Fig. 2:**
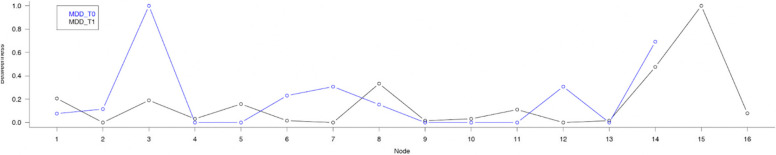
Betweenness index in MDD sample at T0 and T1

As for the centrality analysis, considering particularly "Betweenness” (Fig. 2—blue line), the two nodes with the highest values and thus being the main conduit of information passing within the network are MoCA (3) (1.00) and Interpersonal (14) (0.69). Regarding, indeed, “Strength centrality”, MoCA (3) (1.00) is the node with the highest number of connections, representing the most important node driving the whole network.

Interestingly, nodes assessing affective symptoms such as depression (BDI-II and HDRS) do not have a great influence on the network per se.

At follow up (T1) (Fig. 1—right side), after 12 weeks of pharmacological treatment, the network of MDD patients significantly changes. The network shows less interconnection between nodes (number of edges = 34; density index = 0,28) than T0, providing us with feedback of greater stability (and less tendency to change) once drug treatment is introduced.

Analyzing the measures of centrality, and considering once again the betweenness (Fig. 2—black line), the node with highest value and, hence, the main information transmission pathway within the network is Antidepressants (15) (1.00).

**Fig. 3:**
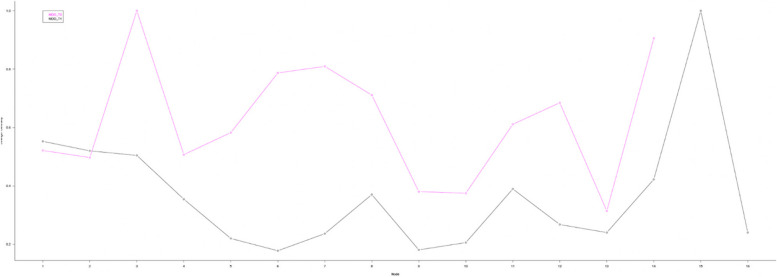
Strength centrality index in MDD sample at T0 and T1

Furthermore, Antidepressants (15) is also the node with the major strength centrality index (1.00) (Fig. 3), representing the node with the highest numbers of connections and, hence, the one that drives the network.

Second-generation antipsychotics play a more peripheral role, in fact, Figs. 2 and 3 show quite low Strength and Betweenness Centrality values. They have far fewer connections and less intensity with the rest of the network than the Antidepressants node. It can be seen, however, that these connections are specific to nodes 4 (FAB),5 (Span_Foreward), and 10 (Fluency_FAS) of the neurocognitive domain and 11 (Autonomy) for the psychosocial domain. Moreover, it is possible to highlight the change in size of the two depressive assessment nodes (1) and (2). This means that the scores have decreased at T1 with the treatment introduction. A decrease of HDRS (1) and BDI-II (2) scoring denotes an improvement in affective symptoms. Despite this, only variance between T0 and T1 for HDRS is statistically significant (T0: *mean* = 23,37; *sd* = 6,90; T1: *mean* = 17,93; *sd* = 9,20; *ANOVA: p* = 0.001).

Additionally, taking into account predictability, HDRS (1) has a percentage of variance explained by the other variables with which it has connections (BDI-II (2) and Antidepressants (15)) equal to 83,2% (R2 = 0.832), while BDI-II (2) has it at 71,5% (R2 = 0.715).

**Fig. 4:**
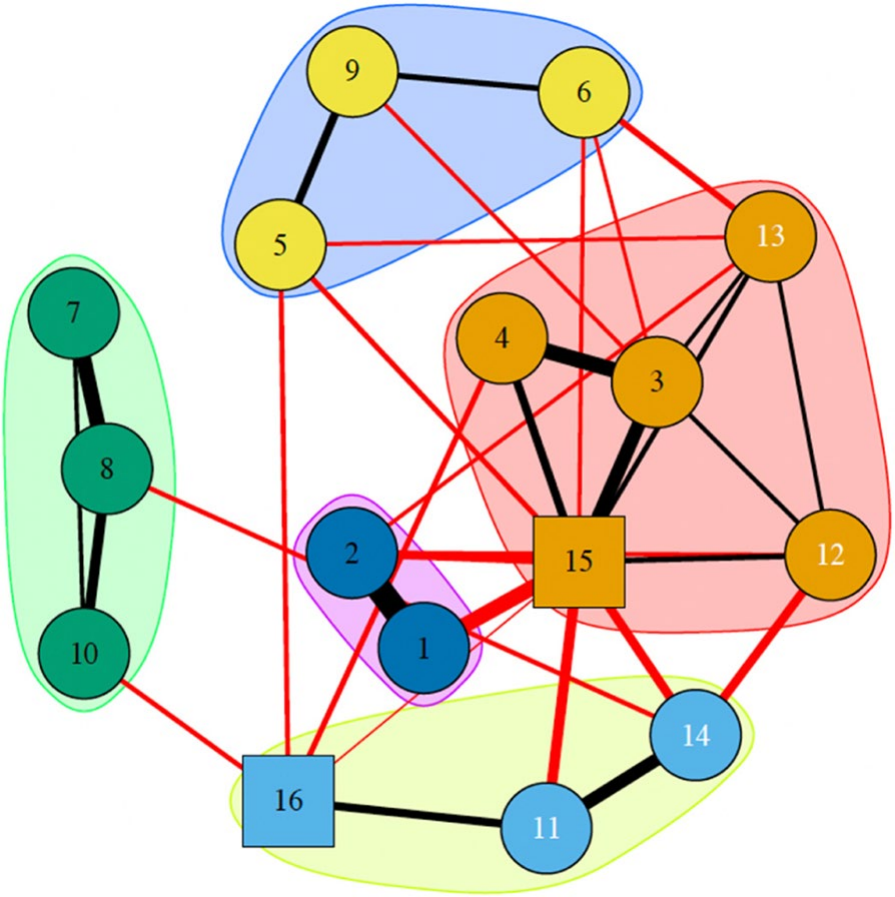
Clusters in MDD sample at T1

Analyzing clusters, the walktrap algorithm used reports the presence of 5 distinct clusters (Fig. 4).

The first, dark blue, includes some neurocognitive variables (5: SPAN_F; 6: SPAN_B; 9: Vocabulary), particularly which involve specifically memory and language. The second, in green, includes other neurocognitive variables (7: REY_I; 8: REY_R; 10: Fluency (FAS)) which also involve memory and language. A third cluster became evident, in purple, including all affective variables. Fourth, an orange cluster is evident, including two neurocognitive variables, in particular global cognitive node (3: MoCA) and executive functioning node (4: FAB), two psychosocial variables, especially cognitive (12) and financial (13), and Antidepressants node (15). Finally, a last cluster has been identified in yellow, including remaining psychosocial variables, such as Autonomy (11) and Interpersonal (14), and Second Generation Antipsychotics (16).

## Discussion

As we already stated and as it is clear from this study, MDD is a complex and heterogeneous mental illness characterized by affective, cognitive, and psychosocial symptoms. Furthermore, these symptoms are closely interlinked between them. Such complexity poses multiple clinical challenges, one of which is certainly the effectiveness of drug treatment. In fact, despite antidepressant drugs such as SSRIs and SNRIs are effective for most depressed patients, 10%-30% of patients with MDD show a partial response to pharmacological treatments and an increased risk of relapse. Recommendations from clinical practice guidelines suggest several strategies to improve the treatment for partial or no responders. These strategies include: adjusting the drugs dosage considering age of patients; concomitant pathological conditions and side effects induced by the antidepressant drug used; or drug replacement and/or augmentation strategies with antipsychotics [20].

In light of this complexity, and to comprehensively understand the multifactorial nature of MDD, several psychometric tools are necessary to catch all its facets. Moreover, the introduction of a new statistical method as NIA can help to identify how symptoms interplay, which of them may be targeted for selective intervention. So, overcoming the limit of low significance that traditional statistical analyses have provided us has been possible.

The purpose of this observational study is to highlight the strengths of the NIA, in order to identify the interaction and changes in network nodes and connections of 14 continuous variables with nodes identified as “Treatment” in MDD.

Analyzing the network of MDD at baseline, neurocognitive and psychosocial domains appear as separated ‘dimensions’ [30]. Despite the whole network is well interlinked, the affective cluster is highly connected with the psychosocial one, and it has very few and weak connections with the cognitive domain. Our data agree with evidence coming from many recent studies suggesting that cognitive dysfunction represents a distinct biological and clinical dimension in MDD, independent from affective symptoms, which strongly affects psychosocial functioning [48–51].

It is also well known that cognitive symptoms could be considered among the most relevant residual symptoms in MDD patients compromising patients working and might predict the low rate of response to antidepressant drugs [52].

In the network at baseline, the key role of cognitive symptoms in MDD is further highlighted by the high strength centrality index of MoCA. It represents the node with the highest number of connections, that drives the whole network. In addition to this, MoCA is the node with the highest betweenness index, that is it is the main conduit and facilitator of information passing within the network. Our results suggest that MoCA might represent a novel and interesting psychometric tool for a better evaluation of cognitive deficits in MDD and to monitor the clinical response to pharmacological and non-pharmacological treatments [14, 53].

When considering the strengths of the NIA method, it is interesting to observe how the network changes after including a 12-week drug treatment as a categorical variable (yellow square). Indeed, when analyzing the network at follow-up, i.e. after the 12 weeks of drug treatment, the network of MDD patients changes significantly. First of all, there is no longer a marked distinction between the cognitive and psychosocial-affective clusters. Additionally, the network shows less interconnection between nodes than T0. According to Cramer et al. (2016) [47] a lower interconnected network is more stable and less vulnerable to change. So greater stability results once drug treatment is introduced with antidepressant drugs.

Pharmacological treatment, especially Antidepressants, take a central role in driving the network. Antidepressants node has the highest betweenness and strength centrality indexes, representing the main information transmission pathway within the network and the most connected node. In contrast to this, Second generation antipsychotics Node remains peripheral in the network, maybe because it has been used as adjunctive pharmacological treatment for partial responders MDD patients. The interesting aspect that the networks represent is that this type of drug interacts primarily with Nodes that represent purely Frontal Functions: short-term memory (SPAN_F), phonemic fluency—FAS (a test that evaluates the ability to generate lists of words, without repeating them, on a phonemic cue; thus, not drawing on long-term memory) and other components tested by means of the Frontal Assessment Battery (FAB). Moreover, this type of medication also acts on the individual's levels of Autonomy (Node 11). In a cognitive assessment of patients, these data must be taken into account in the intra-individual variability of the various time-points.

Moreover, at follow up (T1) it is evident an improvement of affective symptoms, demonstrated by reduction size of HDRS and BDI-II nodes. Along this line, it is interesting to observe that affective nodes are just those with higher levels of predictability. These data suggest that most of the variance of HDRS and BDI-II nodes can be predicted by the variables with which they have direct connections, in this case Antidepressants node.

### Limitations

This study has some limitations: the first limitation concerns the number of patients that completed the follow-up. Given the observational nature of the present study, and the vulnerability of this patients it was problematic to recruit a larger sample, but in future studies it would be essential to enroll a larger number of MDD patients with a recent history of partial response to antidepressants to achieve more relevant results. Despite this, the numerosity of the T1 sample has a discrete power. The last limitation certainly concerns the sampling method, which in this case is non-probabilistic. The patients were all recruited within the same psychiatric clinic unit, which is why the variability of the sample is not so high. Multicentric observational studies might be essential to increase clinical variability.

## Conclusion

To conclude, NIA allows us to understand not only what symptoms enhance after pharmacological treatment, but especially the role it plays within the network and with which nodes it has stronger connections. Moreover, NIA can help identify specific symptoms that may be targeted for intervention, as well as potential pathways for intervention that may have the greatest impact on overall symptom severity. Some scientific papers have found out that NIA could also help to better understand how effective psychotherapy interventions enhance mental disorders symptomatology [24, 25].

NIA represents a promising new approach to understanding and treating complex mental disorders like MDD.

### Supplementary Information

**Additional file 1.**

## Data Availability

The data supporting the findings of the article are available at the follow link: https://figshare.com/s/00ac49c9b681a44aa2e3
