# Supplementary material for: The dynamic interaction between symptoms and pharmacological treatment in patients with major depressive disorder: the role of network intervention analysis
Source: BMC Psychiatry. 2023 Nov 28;23:885. doi: 10.1186/s12888-023-05300-y (PMC10683186; doi:10.1186/s12888-023-05300-y)
Supplement: Supplementary file 1 — Additional file 1. [file 12888_2023_5300_MOESM1_ESM.pdf]

THE DYNAMIC INTERACTION BETWEEN SYMPTOMS AND PHARMACOLOGICAL TREATMENT IN PATIENTS WITH MAJOR DEPRESSIVE DISORDER: THE ROLE OF NETWORK INTERVENTION ANALYSIS

Pierfrancesco Sarti

```
load(file = "Environment.RData")
```

In this document will be is provided the Source Code used to do the Network Analysis (models, predictability, and centrality measures) and Network Representations; as well as the code that generated the images of the Manuscript.

```
MDD_T0 <- as.matrix(MDD_T0)
MDD_T1 <- as.matrix(MDD_T1)

Groups_MDDT0 <- c(rep("Depression",2), rep("Neurocognitive",8),
                  rep("Psychosocial", 4))

Groups_MDDT1 <- c(rep("Depression",2), rep("Neurocognitive",8),
                  rep("Psychosocial", 4), rep("Treatment",2))
```

Creating groups for variable representation of Major Depression

```
net_MDDT0 <- mgm(data = MDD_T0, alphaSel = "CV", alphaFolds = 10,
                type = c("g","g","g","g","g","g","g","g","g",
                         "g","g","g","g","g"),
                level = c("1","1","1","1","1","1","1","1","1",
                          "1","1","1","1","1"),
                k = 2, ruleReg = "AND", alphaSeq = 0, scale = TRUE,
                method = "glm", warnings = FALSE)
```

Mixed graphical Model for Time0 - MDD T0

```
##   |                                                                      |
## Note that the sign of parameter estimates is stored separately; see ?mgm
```

```
qgraph_MDDT0 <- qgraph(net_MDDT0$pairwise$wadj, layout = "spring",
                       edge.color = net_MDDT0$pairwise$edgecolor,
                       groups = Groups_MDDT0, palette = "colorblind",
                       color = c("#0033FF", "#FF66FF","#008000"),
                       nodeNames = colnames(MDD_T0), legend.mode = "style2",
                       legend.cex = 0.4, vTrans = 200,
                       vsize = 5.5, esize = 25)
plot(qgraph_MDDT0)
```

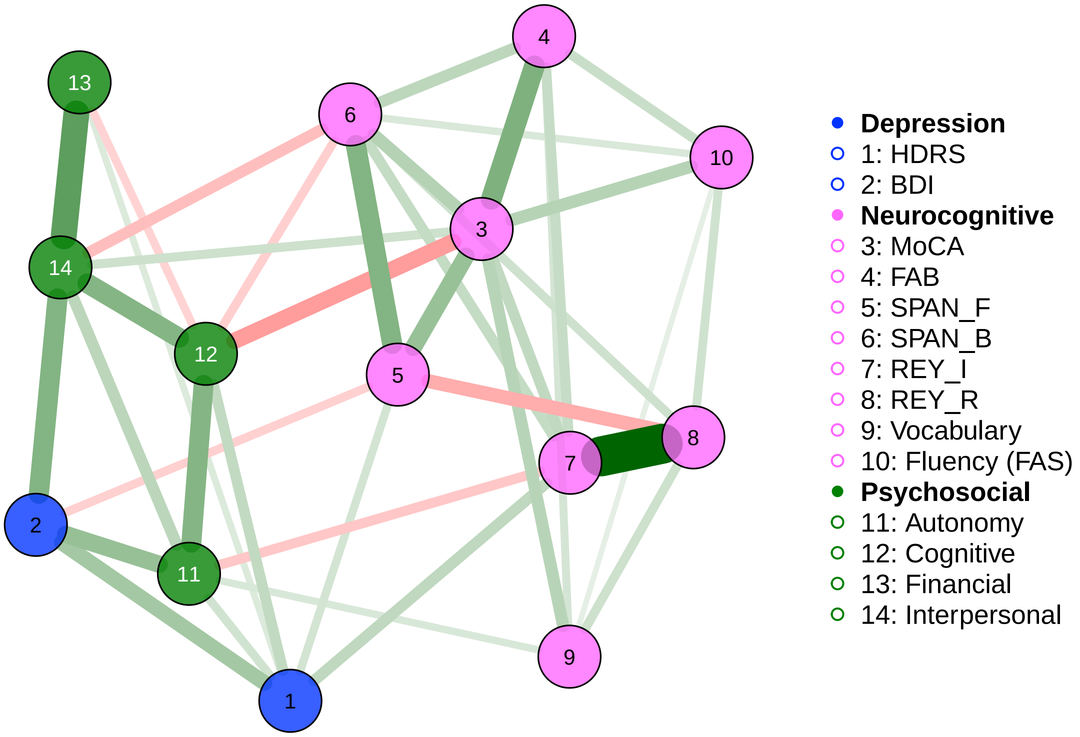


```
net_MDDT1<- mgm(data = MDD_T1, alphaSel = "CV", alphaFolds = 10,
                type = c("g","g","g","g","g","g","g","g","g",
                         "g","g","g","g","g","c","c"),
                level = c("1","1","1","1","1","1","1","1","1",
                          "1","1","1","1","1","2","2"),
                k = 2, ruleReg = "OR", alphaSeq = 0, scale = TRUE,
                method = "glm", warnings = FALSE)
```

Mixed Graphical Model for Time1 - MDD T1

```
##   |                                                                      |
## Note that the sign of parameter estimates is stored separately; see ?mgm
```

```
qgraph_MDDT1 <- qgraph(net_MDDT1$pairwise$wadj, layout = "spring",
                       edge.color = net_MDDT1$pairwise$edgecolor,
                       groups = Groups_MDDT1, palette = "colorblind",
                       color = c("#0033FF", "#FF66FF","#008000", "#FFFF00"),
                       shape = c("circle","circle","circle","circle",
                                 "circle","circle","circle","circle",
                                 "circle","circle","circle","circle",
                                 "circle","circle","square","square"),
                       nodeNames = colnames(MDD_T1), legend.mode = "style2",
                       legend.cex = 0.4, vTrans = 200,
                       vsize = 5.5, esize = 25)
plot(qgraph_MDDT1)
```

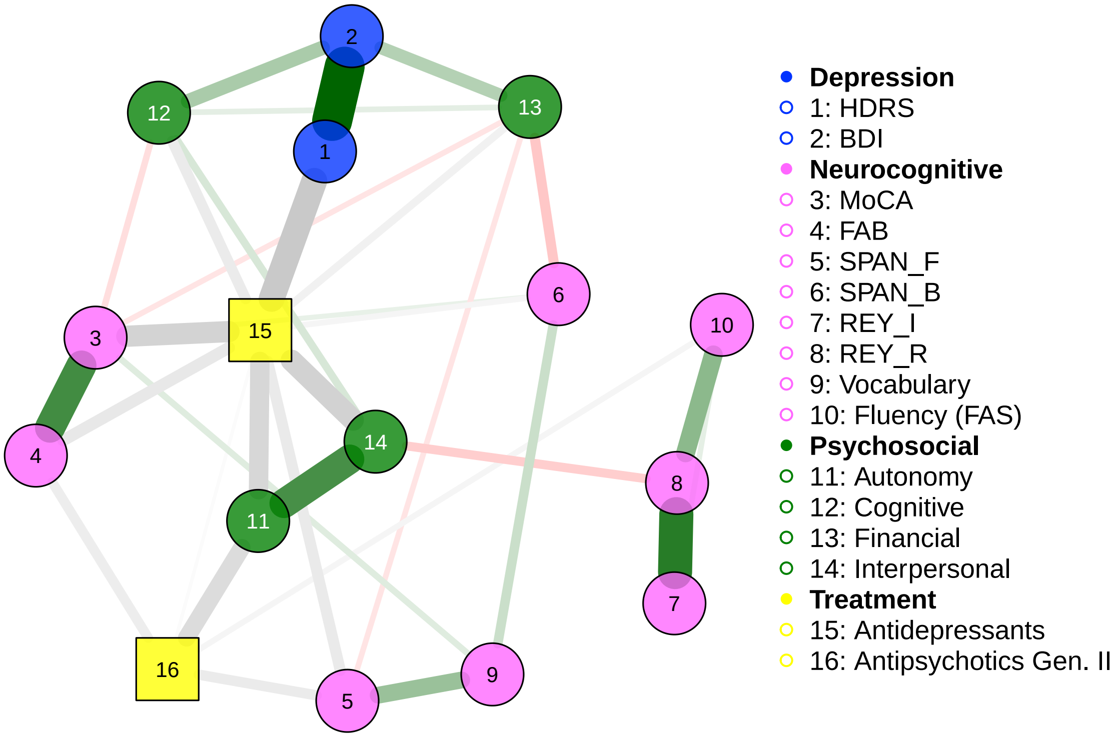


```
A <- ncol(MDD_T0)

pred_objA <- predict(object = net_MDDT0, data = MDD_T0,
                     errorCat = c("CC", "nCC", "CCmarg"),
                     errorCon = c("R2"))
errorsA <- list()
for(i in 1:14) errorsA[[i]] <- pred_objA$errors[i,2]

color_listA <- list()
for(i in 1:14) color_listA[[i]] <- "#000000"
```

Predictability of the Nodes MDD_T0

```
B <- ncol(MDD_T1)

pred_objB <- predict(object = net_MDDT1, data = MDD_T1,
                     errorCat = c("CC", "nCC", "CCmarg"),
                     errorCon = c("R2"))
errorsB <- list()
for(i in 1:14) errorsB[[i]] <- pred_objB$errors[i,2]
for(i in 15:16) errorsB[[i]] <- pred_objB$errors[i,4]

color_listB <- list()
for(i in 1:16) color_listB[[i]] <- "#000000"
```

Predictability of the Nodes MDD_T1

```
NET_MDD_T0 <- qgraph(net_MDDT0$pairwise$wadj,
                     layout = "spring", groups = Groups_MDDT0,
                     vsize = 6, vTrans = 200, theme = "colorblind",
                     edge.color = net_MDDT0$pairwise$edgecolor,
                     nodeNames = colnames(MDD_T0), legend.mode = "style2",
                     legend.mode = "style2", legend.cex = 0.4,
                     color = c("#0033FF", "#FF66FF","#008000"),
                     pie = errorsA, pieColor = color_listA,
                     title = "MDD T0")
```

Representation of the final network of MDD T0 & T1 with predictability rings

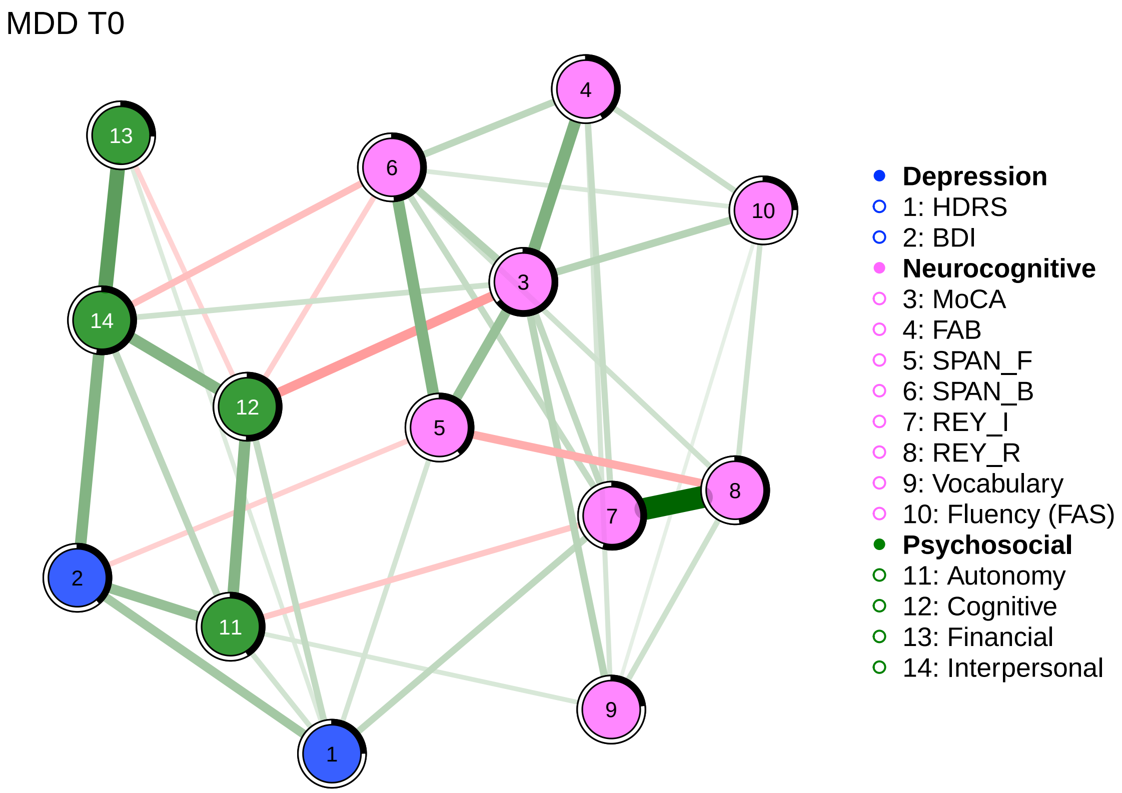


```
NET_MDD_T1 <- qgraph(net_MDDT1$pairwise$wadj,
                     layout = "spring", groups = Groups_MDDT1,
                     vsize = c(3.67,4.59,6.99,6.72,5.86,5.22,6.65,7.36,6.18,
                               5.93,5.83,5.85,6.10,5.85, 6,6),
                     vTrans = 200, theme = "colorblind",
                     edge.color = net_MDDT1$pairwise$edgecolor,
                     nodeNames = colnames(MDD_T1), legend.mode = "style2",
                     legend.mode = "style2", legend.cex = 0.4,
                     color = c("#0033FF", "#FF66FF","#008000", "#FFFF00"),
                     shape = c("circle","circle","circle","circle",
                               "circle","circle","circle","circle",
                               "circle","circle","circle","circle",
```

```
                                  "circle","circle","square","square"),
                        pie = errorsB, pieColor = color_listB,
                        title = "MDD T1")
```

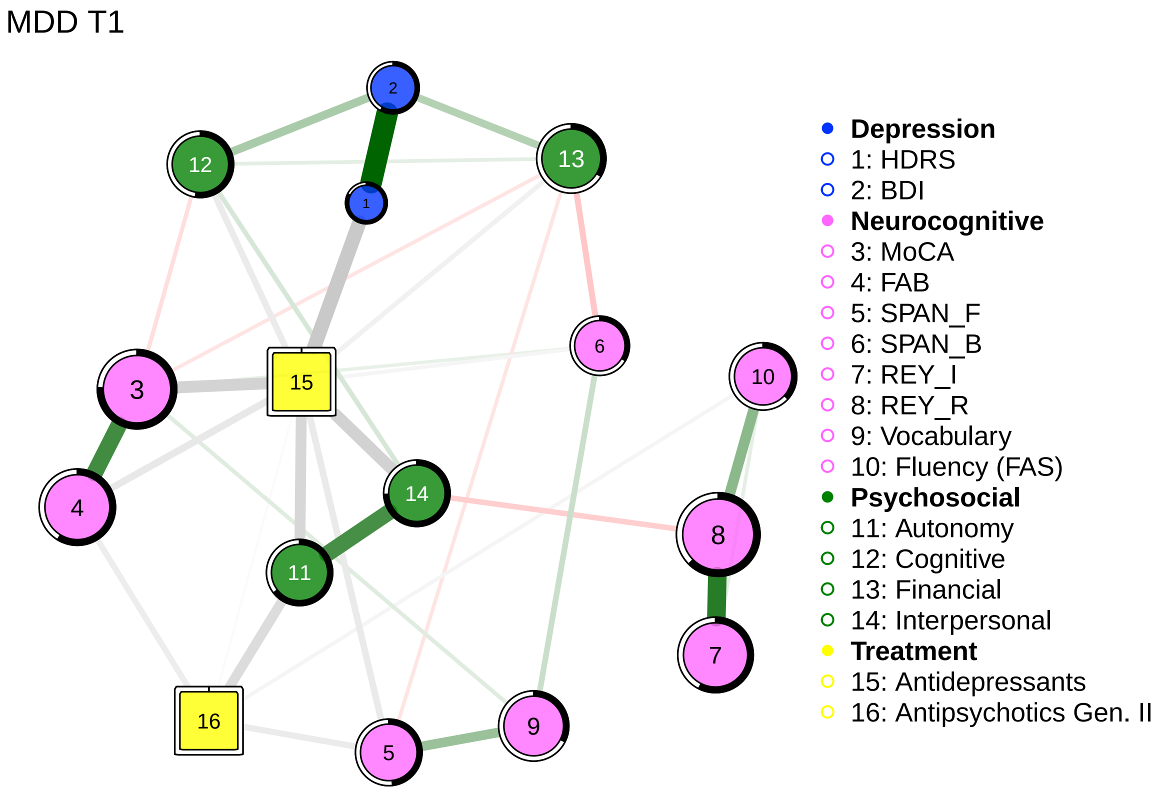

< #### Transformation from qgraph to igraph object to do cluster analysis #### <

```
MDDT0_igraph <- as.igraph(NET_MDD_T0)
MDDT1_igraph <- as.igraph(NET_MDD_T1)
```

< #### Cluster MDD with walktrap algorithm for MDD T0 and MDD T1 in order to see where the there is a specific are where drugs take action #### <

```
walktrap_MDDT0 <- cluster_walktrap(MDDT0_igraph)
plot(walktrap_MDDT0, MDDT0_igraph, layout = NET_MDD_T0$layout)
```

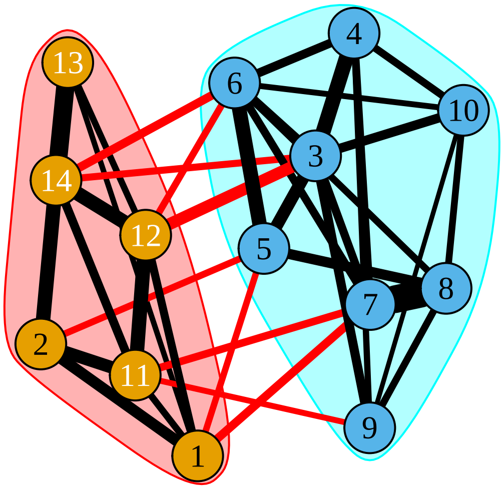


```
walktrap_MDDT1 <- cluster_walktrap(MDDT1_igraph)
plot(walktrap_MDDT1, MDDT1_igraph, layout = NET_MDD_T1$layout)
```

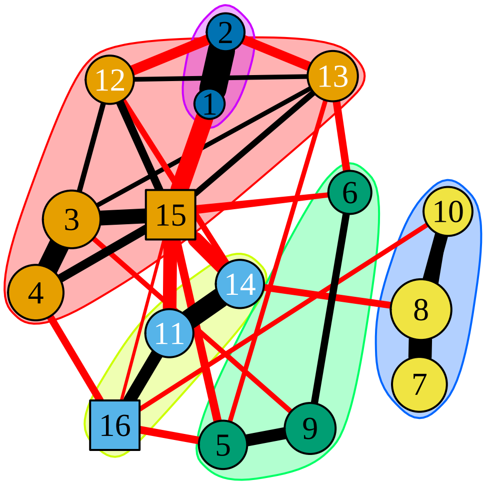


```r
ecount(MDDT0_igraph)
```

Network Density and edge count

```
## [1] 39
```

```r
edge_density(MDDT0_igraph, loops = FALSE)
```

```
## [1] 0.4285714
```

```r
ecount(MDDT1_igraph)
```

```
## [1] 33
```

```r
edge_density(MDDT1_igraph, loops = FALSE)
```

```
## [1] 0.275
```

```r
CentralityPlot <- centralityPlot(NET_MDD_T0,
                                 include = c("Strength","Betweenness"),
                                 scale = "relative", decreasing = TRUE,
                                 theme_bw = FALSE, weighted = TRUE,
                                 labels = colnames(MDD_T0))
```

CENTRALITY ANALISYS

```
## Note: relative centrality indices are shown on x-axis rather than raw centrality indices.
```

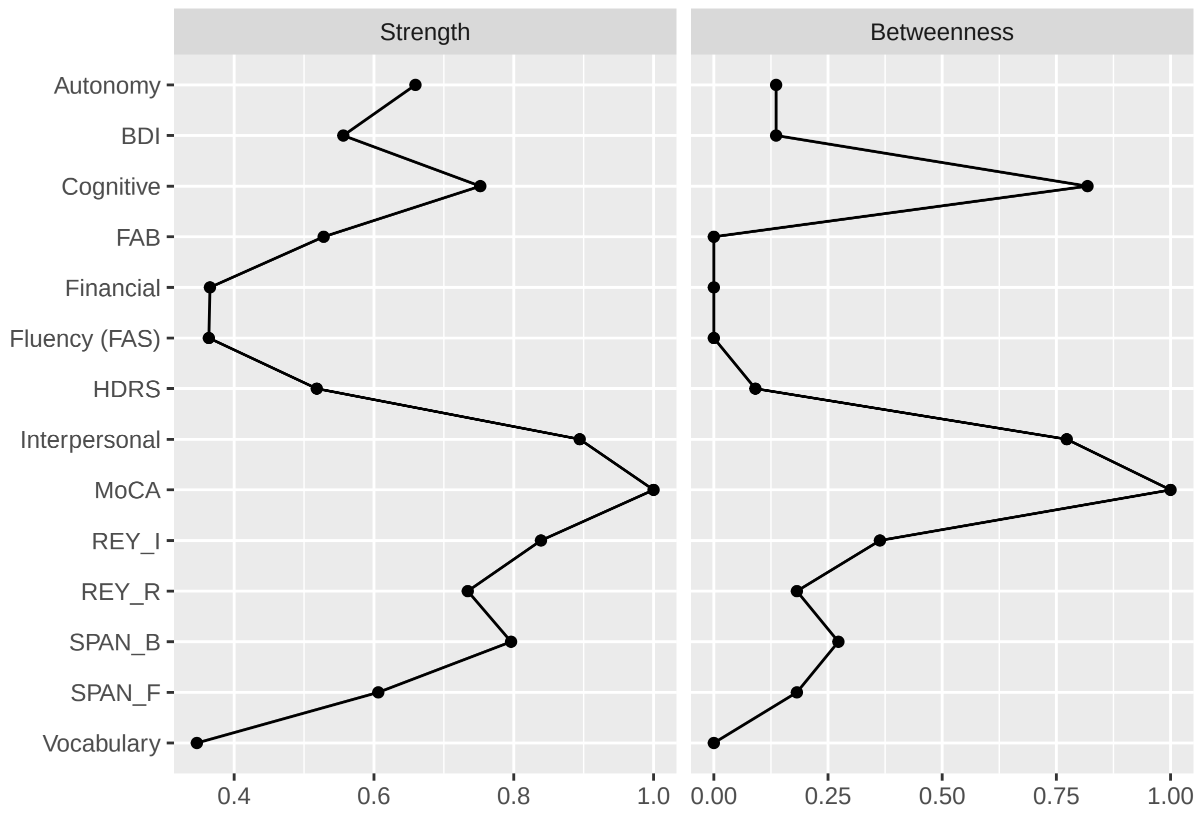

Bootstrap centrality Major Depressive Disorder boot_MDD_T0 <- bootnet(MDD_T0, nBoots = 100, nCores = 4, default = "mgm", type = "case", statistics = c("strength","betweenness", "closeness"))

```
plot(boot_MDD_T0, statistics = c("strength","betweenness","closeness"))
```

```
## Warning in plot.bootnet(boot_MDD_T0, statistics = c("strength", "betweenness",
## : Statistic closeness does not contain any variance and is therefore not shown.
```

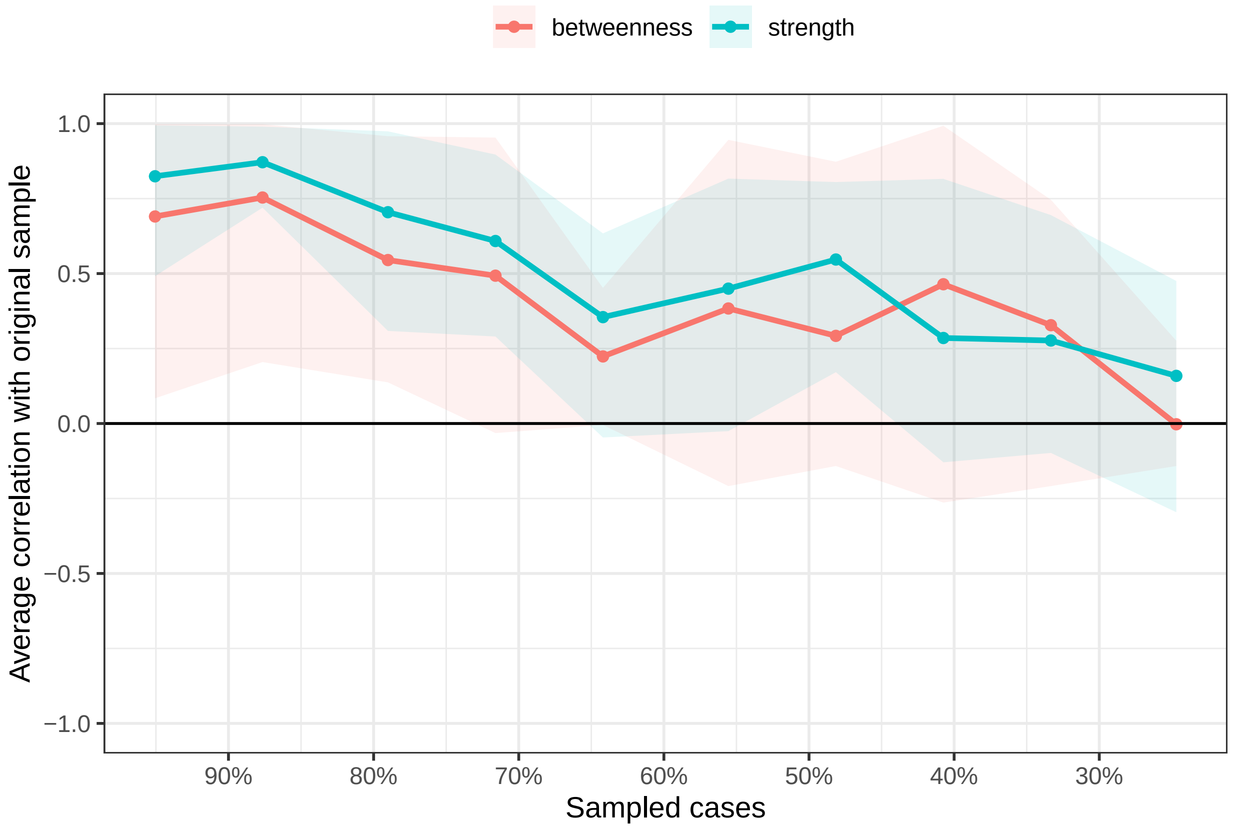

boot_MDD_T1 <- bootnet(MDD_T1, nBoots = 100, nCores = 4, default = “mgm”, type = “case”, statistics = c(“strength”,“betweenness”, “closeness”))

```
plot(boot_MDD_T1, statistics = c("strength","betweenness","closeness"))
```

```
## Warning in plot.bootnet(boot_MDD_T1, statistics = c("strength", "betweenness",
## : Statistic closeness does not contain any variance and is therefore not shown.
```

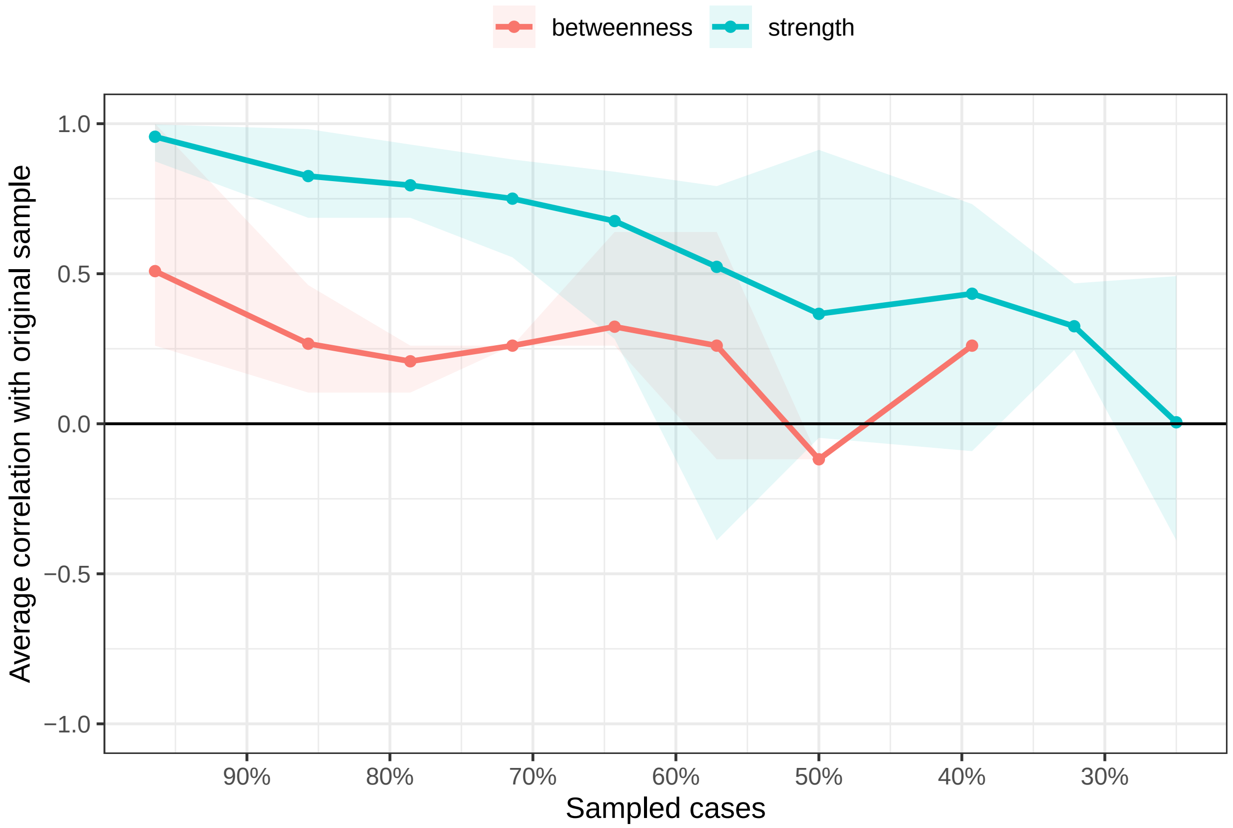


```
cenMDD_T0 <- centralityTable(NET_MDD_T0, standardized = FALSE, relative = TRUE)
cenMDD_T1 <- centralityTable(NET_MDD_T1, standardized = FALSE, relative = TRUE)

cenMDD_T0
```

CUSTOM MADE CENTRALITY PLOT

```
##      graph type node       measure      value
## 1  graph 1   NA    1   Betweenness 0.09090909
## 2  graph 1   NA    2   Betweenness 0.13636364
## 3  graph 1   NA    3   Betweenness 1.00000000
## 4  graph 1   NA    4   Betweenness 0.00000000
## 5  graph 1   NA    5   Betweenness 0.18181818
## 6  graph 1   NA    6   Betweenness 0.27272727
## 7  graph 1   NA    7   Betweenness 0.36363636
## 8  graph 1   NA    8   Betweenness 0.18181818
## 9  graph 1   NA    9   Betweenness 0.00000000
## 10 graph 1   NA   10   Betweenness 0.00000000
## 11 graph 1   NA   11   Betweenness 0.13636364
## 12 graph 1   NA   12   Betweenness 0.81818182
## 13 graph 1   NA   13   Betweenness 0.00000000
## 14 graph 1   NA   14   Betweenness 0.77272727
## 15 graph 1   NA    1     Closeness 0.68467243
## 16 graph 1   NA    2     Closeness 0.73844121
## 17 graph 1   NA    3     Closeness 1.00000000
```

```
## 18 graph 1   NA    4         Closeness 0.75691285
## 19 graph 1   NA    5         Closeness 0.85746847
## 20 graph 1   NA    6         Closeness 0.81931316
## 21 graph 1   NA    7         Closeness 0.79430924
## 22 graph 1   NA    8         Closeness 0.73581575
## 23 graph 1   NA    9         Closeness 0.61757337
## 24 graph 1   NA   10         Closeness 0.60658203
## 25 graph 1   NA   11         Closeness 0.80238607
## 26 graph 1   NA   12         Closeness 0.92800168
## 27 graph 1   NA   13         Closeness 0.63403337
## 28 graph 1   NA   14         Closeness 0.80665358
## 29 graph 1   NA    1          Strength 0.51822035
## 30 graph 1   NA    2          Strength 0.55614186
## 31 graph 1   NA    3          Strength 1.00000000
## 32 graph 1   NA    4          Strength 0.52805484
## 33 graph 1   NA    5          Strength 0.60617965
## 34 graph 1   NA    6          Strength 0.79616162
## 35 graph 1   NA    7          Strength 0.83891893
## 36 graph 1   NA    8          Strength 0.73420235
## 37 graph 1   NA    9          Strength 0.34663214
## 38 graph 1   NA   10          Strength 0.36381765
## 39 graph 1   NA   11          Strength 0.65934275
## 40 graph 1   NA   12          Strength 0.75212558
## 41 graph 1   NA   13          Strength 0.36538777
## 42 graph 1   NA   14          Strength 0.89434855
## 43 graph 1   NA    1 ExpectedInfluence 0.51822035
## 44 graph 1   NA    2 ExpectedInfluence 0.55614186
## 45 graph 1   NA    3 ExpectedInfluence 1.00000000
## 46 graph 1   NA    4 ExpectedInfluence 0.52805484
## 47 graph 1   NA    5 ExpectedInfluence 0.60617965
## 48 graph 1   NA    6 ExpectedInfluence 0.79616162
## 49 graph 1   NA    7 ExpectedInfluence 0.83891893
## 50 graph 1   NA    8 ExpectedInfluence 0.73420235
## 51 graph 1   NA    9 ExpectedInfluence 0.34663214
## 52 graph 1   NA   10 ExpectedInfluence 0.36381765
## 53 graph 1   NA   11 ExpectedInfluence 0.65934275
## 54 graph 1   NA   12 ExpectedInfluence 0.75212558
## 55 graph 1   NA   13 ExpectedInfluence 0.36538777
## 56 graph 1   NA   14 ExpectedInfluence 0.89434855
```

```
cenMDD_T1
```

```
##      graph type node           measure      value
## 1  graph 1   NA    1       Betweenness 0.29850746
## 2  graph 1   NA    2       Betweenness 0.20895522
## 3  graph 1   NA    3       Betweenness 0.19402985
## 4  graph 1   NA    4       Betweenness 0.01492537
## 5  graph 1   NA    5       Betweenness 0.16417910
## 6  graph 1   NA    6       Betweenness 0.01492537
## 7  graph 1   NA    7       Betweenness 0.00000000
## 8  graph 1   NA    8       Betweenness 0.38805970
## 9  graph 1   NA    9       Betweenness 0.02985075
## 10 graph 1   NA   10       Betweenness 0.00000000
## 11 graph 1   NA   11       Betweenness 0.11940299
## 12 graph 1   NA   12       Betweenness 0.00000000
```

```
## 13 graph 1   NA   13       Betweenness 0.04477612
## 14 graph 1   NA   14       Betweenness 0.52238806
## 15 graph 1   NA   15       Betweenness 1.00000000
## 16 graph 1   NA   16       Betweenness 0.00000000
## 17 graph 1   NA    1         Closeness 0.85443577
## 18 graph 1   NA    2         Closeness 0.78093551
## 19 graph 1   NA    3         Closeness 0.76389986
## 20 graph 1   NA    4         Closeness 0.65281576
## 21 graph 1   NA    5         Closeness 0.65855102
## 22 graph 1   NA    6         Closeness 0.44667176
## 23 graph 1   NA    7         Closeness 0.48100814
## 24 graph 1   NA    8         Closeness 0.54012449
## 25 graph 1   NA    9         Closeness 0.53025901
## 26 graph 1   NA   10         Closeness 0.44439972
## 27 graph 1   NA   11         Closeness 0.82746094
## 28 graph 1   NA   12         Closeness 0.60428164
## 29 graph 1   NA   13         Closeness 0.55801238
## 30 graph 1   NA   14         Closeness 0.87941539
## 31 graph 1   NA   15         Closeness 1.00000000
## 32 graph 1   NA   16         Closeness 0.68757112
## 33 graph 1   NA    1          Strength 0.51001430
## 34 graph 1   NA    2          Strength 0.50920724
## 35 graph 1   NA    3          Strength 0.53217758
## 36 graph 1   NA    4          Strength 0.38319922
## 37 graph 1   NA    5          Strength 0.30679318
## 38 graph 1   NA    6          Strength 0.19645120
## 39 graph 1   NA    7          Strength 0.29721390
## 40 graph 1   NA    8          Strength 0.46590143
## 41 graph 1   NA    9          Strength 0.22818190
## 42 graph 1   NA   10          Strength 0.20732068
## 43 graph 1   NA   11          Strength 0.49906056
## 44 graph 1   NA   12          Strength 0.30086994
## 45 graph 1   NA   13          Strength 0.30977658
## 46 graph 1   NA   14          Strength 0.49886905
## 47 graph 1   NA   15          Strength 1.00000000
## 48 graph 1   NA   16          Strength 0.31573258
## 49 graph 1   NA    1 ExpectedInfluence 0.51001430
## 50 graph 1   NA    2 ExpectedInfluence 0.50920724
## 51 graph 1   NA    3 ExpectedInfluence 0.53217758
## 52 graph 1   NA    4 ExpectedInfluence 0.38319922
## 53 graph 1   NA    5 ExpectedInfluence 0.30679318
## 54 graph 1   NA    6 ExpectedInfluence 0.19645120
## 55 graph 1   NA    7 ExpectedInfluence 0.29721390
## 56 graph 1   NA    8 ExpectedInfluence 0.46590143
## 57 graph 1   NA    9 ExpectedInfluence 0.22818190
## 58 graph 1   NA   10 ExpectedInfluence 0.20732068
## 59 graph 1   NA   11 ExpectedInfluence 0.49906056
## 60 graph 1   NA   12 ExpectedInfluence 0.30086994
## 61 graph 1   NA   13 ExpectedInfluence 0.30977658
## 62 graph 1   NA   14 ExpectedInfluence 0.49886905
## 63 graph 1   NA   15 ExpectedInfluence 1.00000000
## 64 graph 1   NA   16 ExpectedInfluence 0.31573258
```

FIGURE 2 - Betweenness Centrality MDD-T0 and MDD-T1 pdf("CustomBetweenness_MDDNetwork.pdf", width = 20, height = 5)

```
plot(x <- cenMDD_T1$value[1:16],
     type = "b",
     bty = "l",
     family = "sans",
     las = 1, lwd = 1,
     xaxt = "n",
     xlab = "Node",
     ylab = "Betweenness")
axis(side = 1, labels = c(1:16), at = c(1:16))
lines(cenMDD_T0$value[1:14], type = "b", col = "#0000FF")
legend(x = 1, legend = c("MDD_T0", "MDD_T1"),
       text.col = c("#0000FF","#000000"))
```

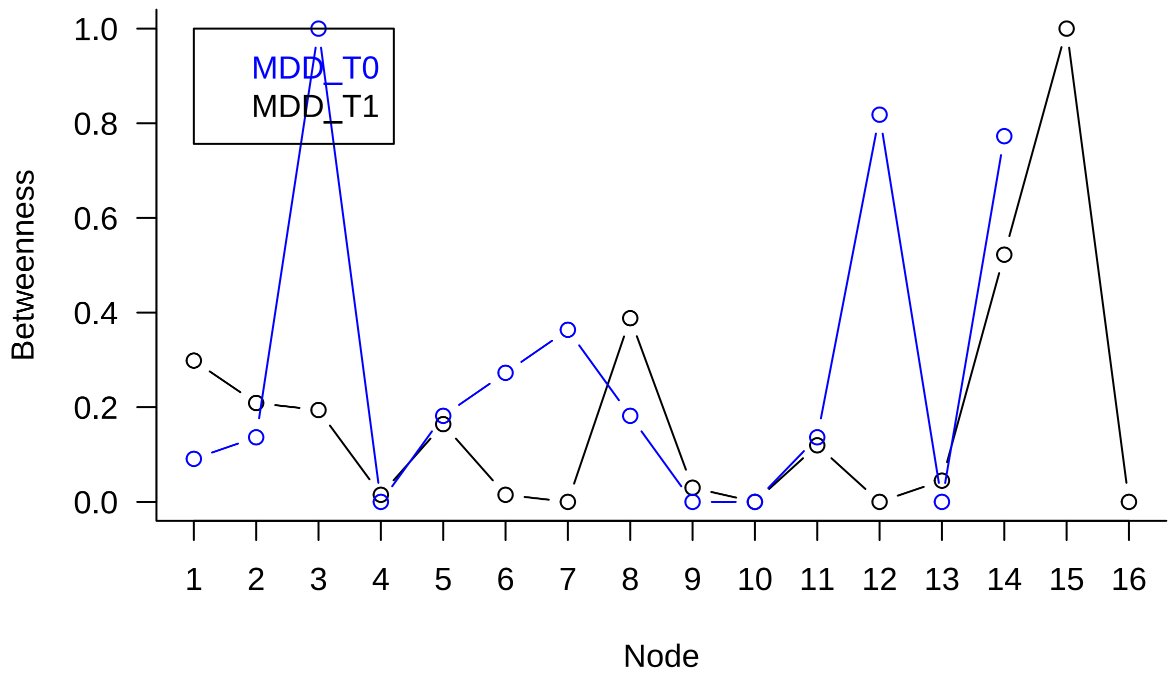

dev.off()

FIGURE 3 - Strength Centrality MDD pdf("CustomStrength_MDDNetwork.pdf", width = 40, height = 15)

```
plot(cenMDD_T1$value[33:48],
     type = "b",
     bty = "l",
     family = "sans",
     las = 1, lwd = 1,
     xaxt = "n",
```

```
     xlab = "Node",
     ylab = "Strength Centrality")
axis(side = 1, labels = c(1:16), at = c(1:16))
lines(cenMDD_T0$value[29:42], type = "b", col = "#FF00FF")
legend(x = 1, legend = c("MDD_T0", "MDD_T1"),
       text.col = c("#FF00FF","#000000"))
```

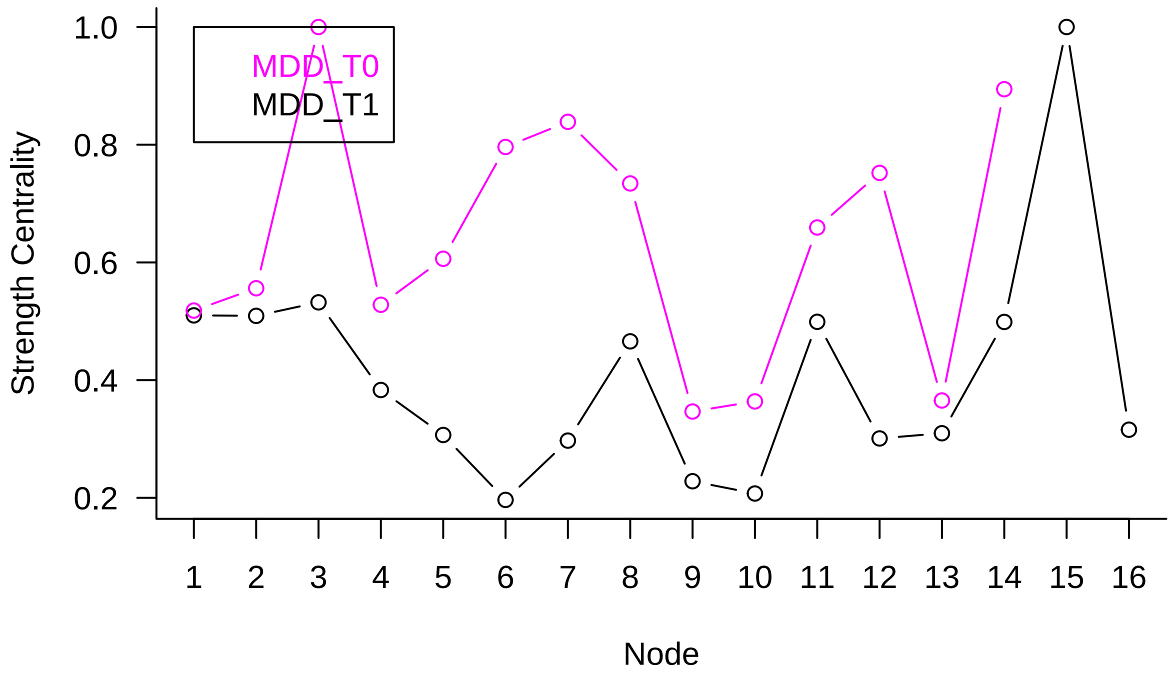

dev.off()
